# Data set for estimating combining abilities for yield and quality attributes in summer tomato using line by tester analysis in Bangladesh

**DOI:** 10.1016/j.dib.2024.111063

**Published:** 2024-10-31

**Authors:** Mohammad Matin Akand, Mohammed Abu Taher Masud, Md. Azizul Hoque, Mohammad Mostafa Kamal, Mohammad Rezaul Karim, Bahauddin Ahmed

**Affiliations:** a Bangladesh Agricultural Research Institute, Gazipur, 1701, Bangladesh; b Bangabandhu Sheikh Mujibur Rahman Agricultural University, Salna, Gazipur, 1706, Bangladesh

**Keywords:** Summer tomato, Hybrid, Combining abilities, Line × Tester analysis, R-statistical software, Agricolae package

## Abstract

This article provides a dataset for line × tester analysis in the F1 generation of summer tomatoes using open-source R statistical software and the ‘agricolae’ package. The dataset includes seven inbred lines as female parents (L) and two testers as male parents (T) with diverse genetic bases and heat tolerance qualities. Fourteen cross combinations were produced through L × T (7 × 2) mating design, involving hybridization between lines (*f*) and testers (*m*) in a one-to-one fashion. To assess the heterosis of the crosses, all parents (both lines and testers) were included along with the crosses and evaluated in the same experimental field for 16 traits using a randomized complete block design (RCBD) with two replications. The line × tester analysis estimates the ANOVA, including parents, combining ability, genetic components, and the contribution of parental lines to genetic variation in the hybrids. This dataset is valuable for breeders in subtropical countries to develop efficient breeding strategies for hybrid summer tomato varieties.

Specifications Table

**Table:** 

Subject	Agricultural Science, Horticulture, Genetics, Data Mining and Statistical Analysis.
Specific subject area	Development of summer tomato hybrid through L × T (7 × 2) mating design, involving hybridization between lines (f) and testers (m) in a one-to-one fashion.
Type of data	Raw, Analyzed, Table, Figure
Data collection	Seeds of the lines, testers, and their hybrids were sown in a well-prepared seedbed. Forty-day-old tomato seedlings were then transplanted into the main field under transparent polytunnels. The polytunnels were 2.3 meters wide and contained two-unit beds, each measuring 0.8 meters by 1 meter, with a 30-centimeter drain between the 14-unit beds. Each unit bed had double rows, accommodating 24 plants. Most of the data were collected from randomly selected plants—five plants per parental line and their crosses. Data collection followed the standards outlined by the International Plant Genetic Resources Institute (IPGRI) descriptor for tomato. Fruits per plant, yield per plant, and yield per hectare were calculated from the plot yield.
Data source location	The trial was implemented in the polytunnels of the Horticulture Research Centre (HRC) of Bangladesh Agricultural Research Institute (BARI), Gazipur-1701, Bangladesh (23°59′27.7"N 90°24′42.4"E, 8.4 masl)
Data accessibility	Repository name: Mendeley Data identification number: 10.17632/nmbhxxxzzj.1 Direct URL to data: https://data.mendeley.com/datasets/nmbhxxxzzj/1
Related research article	Akand, M.M., Kamal, M.M., Haque, M.I., Brahma, S., Yousuf, M.N., & Khatun, M. (2024). A dataset on multi-trait selection approach for the evaluation of F1 tomato hybrids along with their parents under hot and humid conditions in Bangladesh. *Data in Brief, 56*, 110859. https://doi.org/10.1016/j.dib.2024.110859.

## 1 Value of the Data

• The dataset from the Line × Tester analysis serves as a rapid screening tool for genetic stocks based on general combining ability (GCA) and specific combining ability (SCA) effects, rather than their variances. This approach can lead to more efficient use of resources in breeding programs.
• Improved hybrid varieties can increase productivity and profitability for farmers. Better-performing hybrids can result in higher market prices and reduced losses due to pests and diseases.
• The dataset supports the development of tomato varieties better adapted to changing climatic conditions, contributing to sustainable and best agronomic practices.
• It creates a platform that facilitates easy sharing, reuse, and collaboration on datasets, especially for researchers working on hybrid development of summer tomatoes or similar agricultural projects.

## 2 Background

Tomatoes play a crucial role in Bangladeshi cuisine, with high year-round demand [1], yet yields are stuck by the shortage of high-yielding varieties and the crop's sensitivity to the hot [2] and wet summer climate. Research conducted at the Horticulture Research Centre in Bangladesh evaluated various tomato germplasms during both winter and summer seasons, highlighting significant variability in yield, fruit characteristics, and pest infestation [3,4]. Line × Tester analysis dataset addresses the need for high-yielding and disease-resistant summer tomatoes. By extending the growing season beyond winter, summer tomato cultivation can stabilize prices and support better nutrition and food security. The dataset was generated to evaluate the combining ability of various parental lines and their crosses to identify hybrids suitable for challenging summer conditions. This approach is grounded in enhancing crop genetics through hybridization. Combining ability analysis is a powerful tool for selecting desirable parent combinations to exploit heterosis or hybrid vigour [[5], [6], [7], [8]]. In the context of the related research article, this dataset provides empirical data supporting the selection of high-performing tomato hybrids. It helps identify specific parent combinations that exhibit the best traits for summer cultivation, contributing to the development of stable, high-yielding, and disease-resistant tomato varieties. This work is crucial for extending its growing season and improving the livelihoods of tomato growing farmers in Bangladesh*.*

## 3 Data Description

**Fig. 1:**
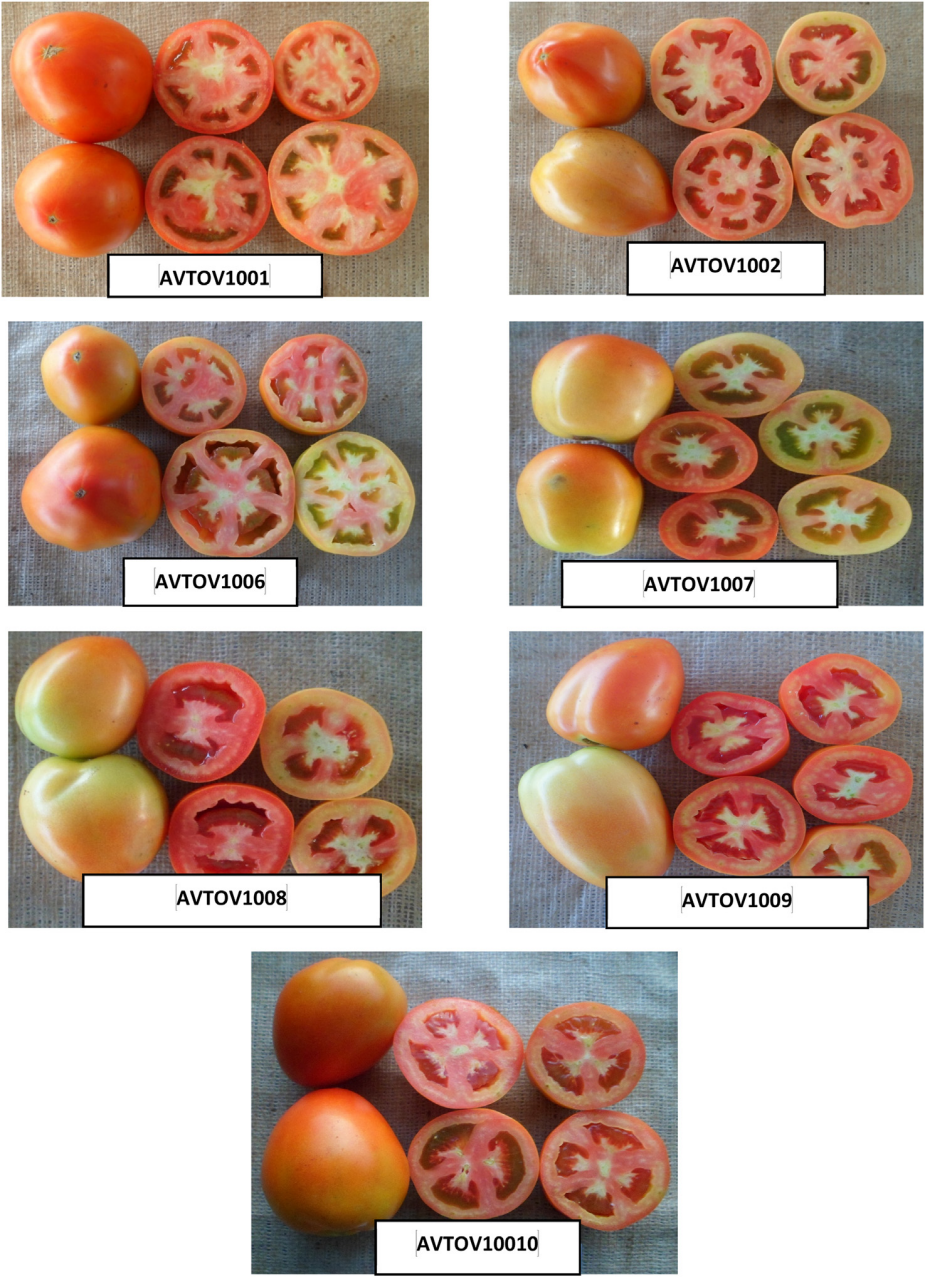
Fruits of lines of summer tomato involved in crossing.

**Fig. 2:**
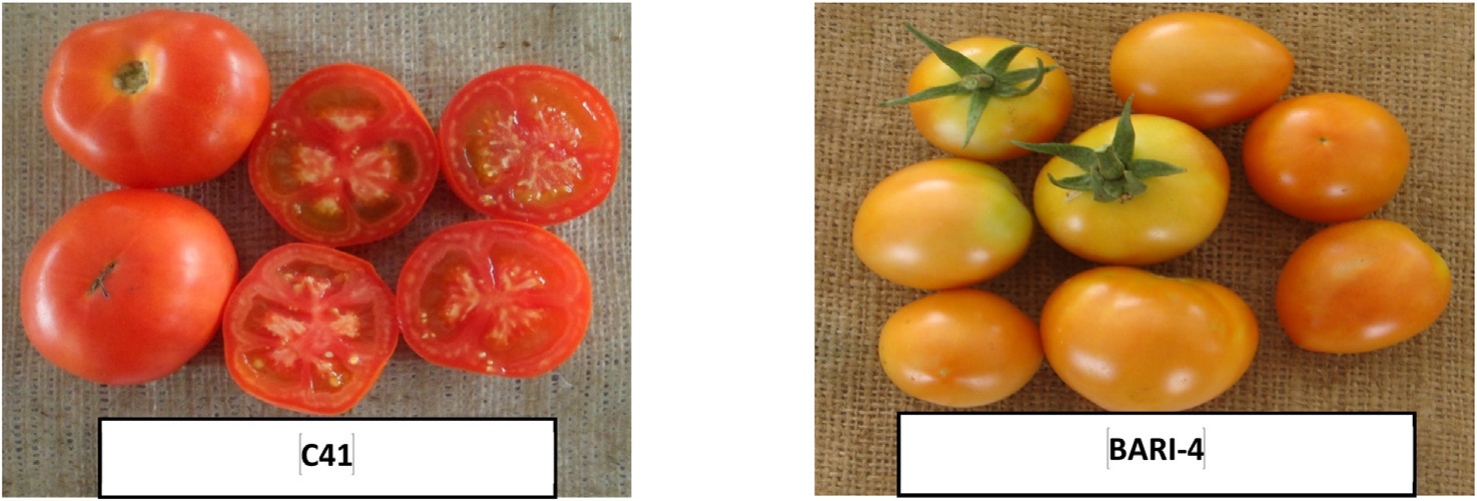
Fruits of the testers of summer tomato involved in crossing.

The dataset presented in this article includes three figures and five tables. Analysis of variance based on the mean square of different characters in summer tomato is presented in Table 1a, Table 1b. The photograph of fruits of the seven lines, two testers and 14 cross combinations, synthesis from them are presented in Figs. 1, 2 and (Fig. 3a & b) respectively. The proportional contribution of lines, testers and their interaction for all the studied traits are presented in Table 2. The estimation of both heritability and genetic advance are presented in Table 3. Estimates of the general combining ability (gca) effects of seven parents and specific combining ability (sca) effects of fourteen hybrids for all the measured characters sin this study are presented in (Tables 4a & b) and 5a & b)*.*

**Table 1a:** Analysis of variance based on mean squares and estimates of the genetic component in line × tester study of summer tomato.

Source	d.f	Plant height at last harvest	No. of branches per plant	Days to 50% flowering	No. of flowers per cluster	No. of fruits per cluster	Fruit length	Fruit width	Fruit shape index
Replication	1	23.39	11.50*	1.07	0.10	0.15	0.07	0.03	1.96
Treatment	22	1332.98**	5.61*	57.65**	1.13**	0.74*	1.06**	0.69**	2.57**
Parents	8	1859.04**	7.18*	70.47**	0.74	0.58	1.89**	1.19**	3.72**
Parent vs. Crosses	1	9060.50**	38.69**	646.67**	9.13**	6.55**	5.01**	1.55**	3.55**
Crosses	13	414.83	2.11	4.45**	0.76	0.38	0.24	0.33**	1.78**
Lines	6	355.16	2.01	4.48	1.22*	0.54	0.24	0.60*	2.96**
Testers	1	212.30	3.29	3.57	1.18	0.66	0.95*	0.003	4.89**
Lines × Testers	6	508.24	2.02	4.57**	0.23	0.18	0.128	0.10	7.81
Error	22	229.81	2.59	0.79	0.39	0.32	0.14	0.06	4.29

σ ^2^gca (Line)		−38.27	−1.11	−0.02	0.25	0.09	0.03	0.13	0.007
σ ^2^gca (Tester)		−21.14	0.09	−0.07	0.07	0.03	0.06	−0.007	0.003
σ ^2^gca (Aveg.)		−4.57	0.005	−0.01	0.03	0.01	0.005	0.01	0.0008
σ ^2^sca (Aveg.)		64.38	−0.07	1.71	0.24	0.07	0.15	0.09	0.01
σ ^2^gca/σ ^2^sca		−0.071	−0.071	−0.006	0.125	0.143	0.033	0.111	0.080
σ ^2^A		−9.13	0.009	−0.01	0.05	0.019	0.01	0.02	0.001
σ ^2^D		139.22	−0.29	1.89	−0.08	−0.07	−0.01	0.02	−0.001
(σ ^2^D/ σ ^2^A)^1/2^		3.9	5.7	13.7	1.3	1.9	1.0	1.0	1.0

*, ** significant at 5 and 1% levels of probability, respectively.

**Table 1b:** Analysis of variance based on mean squares and estimates of the genetic component in line × tester study of summer tomato.

Source	d.f	No. of locules per fruit	Average fruit weight	No. of fruits per plant	Yield per plant	Yield per hectare	Total soluble solids	TYLCV incidence	Bacterial wilt incidence
Replication	1	0.45	1.23	2.13	0.0005	0.57	0.01	74.04*	27.18
Treatment	22	1.94**	2041.38**	315.74**	0.7947**	163.90**	0.89**	35.27**	76.07**
Parents	8	2.23**	3718.24**	43.67**	0.1549**	53.05**	0.88**	21.02	15.15
Parent vs. Crosses	1	7.08**	11291.61**	4324.18**	7.3565**	2907.95**	0.80**	238.67**	50.58**
Crosses	13	1.36**	297.91**	174.83**	0.6837**	21.04**	0.91**	28.40*	61.67**
Lines	6	2.66**	473.02	149.28	0.9507	30.72	0.66	13.51	71.06
Testers	1	1.24**	186.79	131.98	0.7394	16.48	2.95	147.29*	7.92
Lines × Testers	6	0.08	141.32**	207.52**	0.4075**	12.12**	0.81**	23.47	44.57
Error	22	0.11	2.66	3.54	0.0011	0.80	0.10	11.52	16.28

σ ^2^gca (Line)		0.65	82.93	−14.56	0.1358	4.65	−0.04	−2.49	6.62
σ ^2^gca (Tester)		0.08	3.25	−5.40	0.0237	0.31	0.15	8.84	4.52
σ ^2^gca (Aveg.)		0.06	7.65	−1.60	0.0135	0.44	0.00	0.24	0.84
σ ^2^sca (Aveg.)		0.6	132.19	79.69	0.3490	9.49	0.69	24.95	29.12
σ ^2^gca/σ ^2^sca		0.10	0.06	−0.02	0.0387	0.05	0.00	0.01	0.03
σ ^2^A		0.13	15.31	−3.20	0.0270	0.87	0.01	0.48	1.67
σ ^2^D		−0.015	69.33	101.99	0.2032	5.66	0.36	5.97	14.14
(σ ^2^A/ σ ^2^D)^1/2^		0.3	2.1	5.6	0.36	2.6	6.0	3.5	2.9

* and **: Indicates significant at 1 and 5% level of probability, respectively.

**Fig. 3a:**
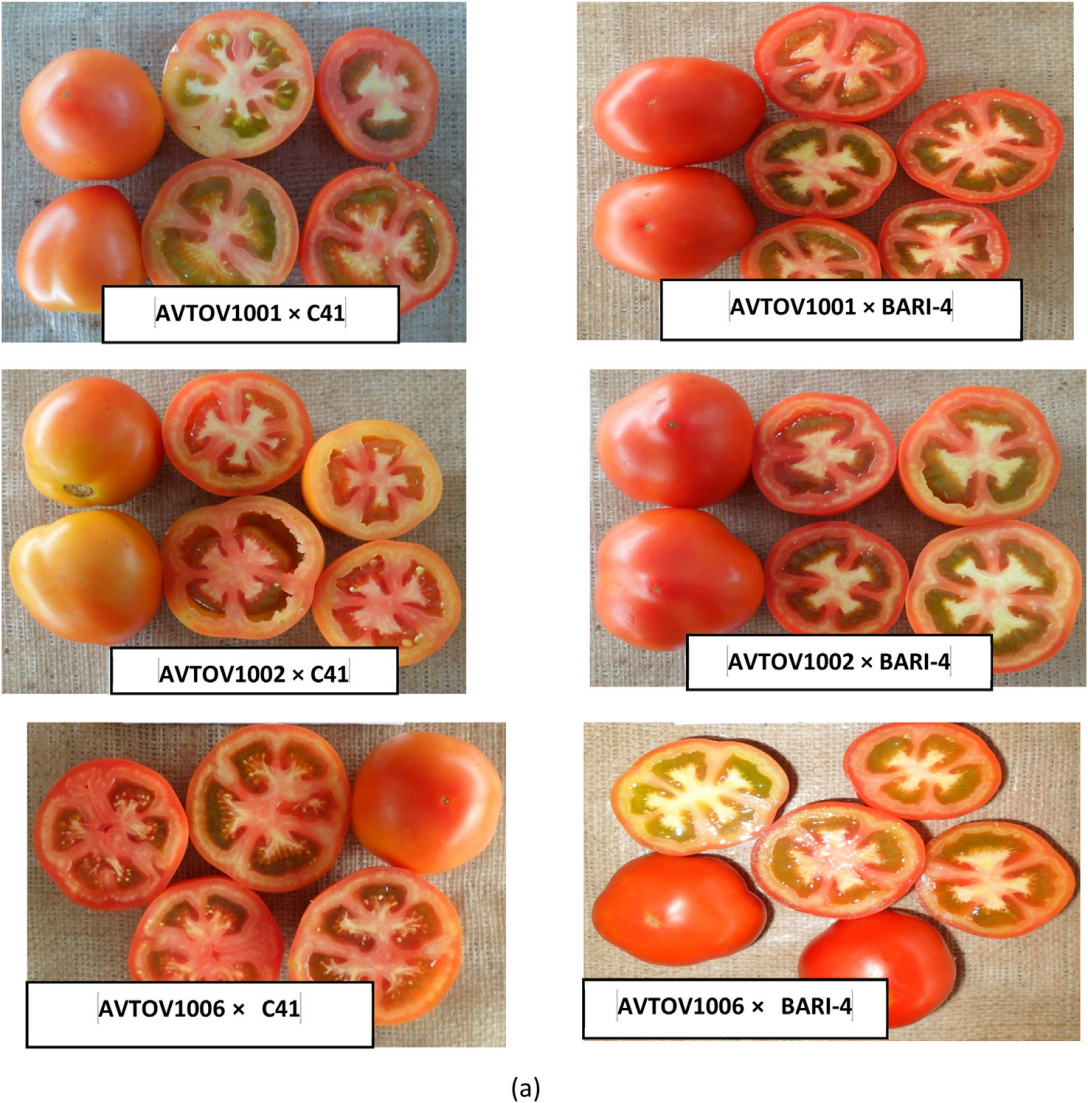
Fruits of summer tomato hybrids.

**Fig. 3b:**
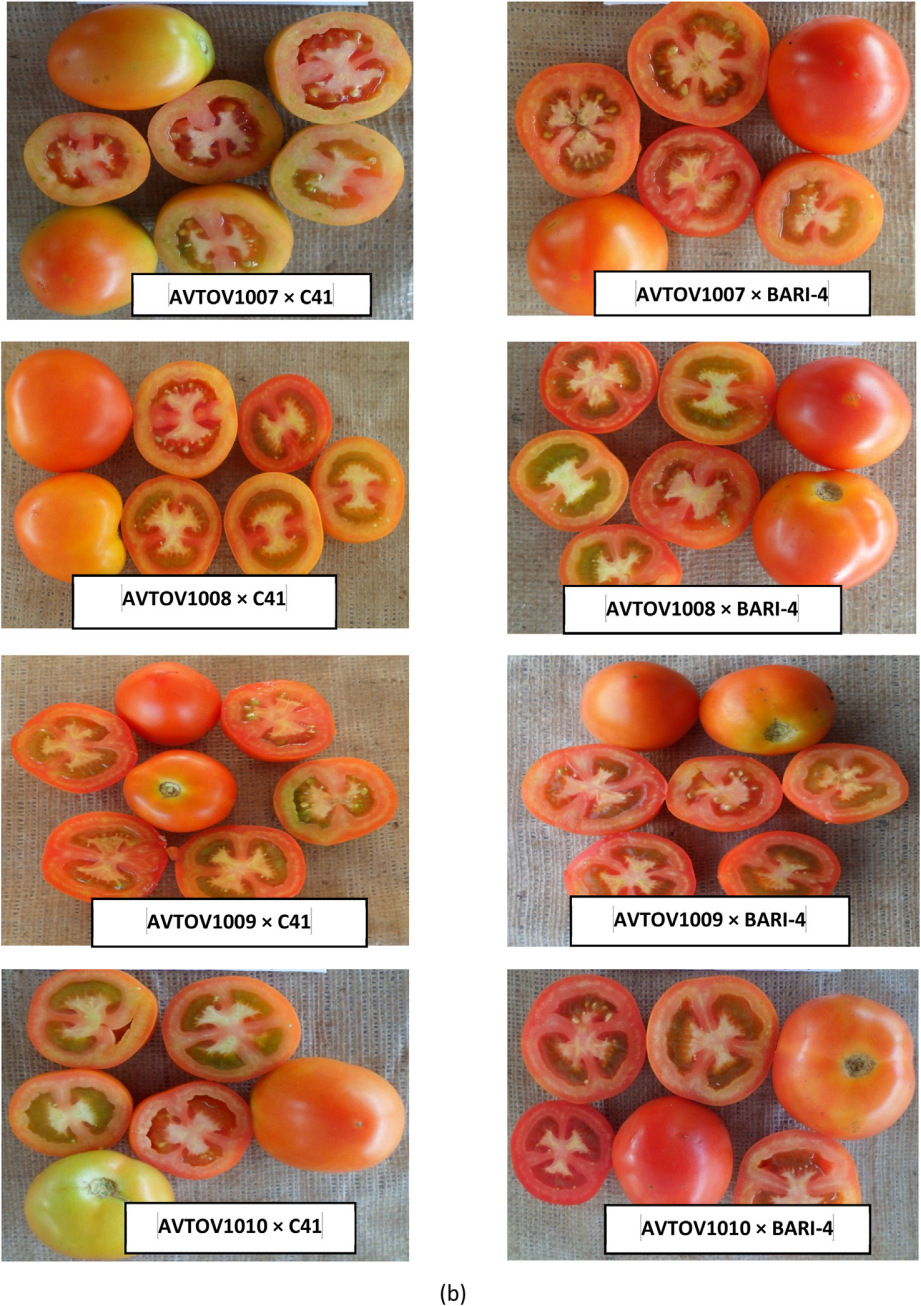
Fruits of summer tomato hybrids.

**Table 2:** Proportional contribution (%) of lines, testers and lines × testers to total variance of summer tomato.

Sl. No.	Characters	Per cent contribution of		
		Lines	Testers	Lines × Testers
1.	Plant height at last harvest	39.52	3.94	56.55
2.	Number of branches per plant	44.02	11.97	44.02
3.	Days to 50% flowering	46.42	6.17	47.41
4.	Number of flowers per cluster	73.97	11.94	14.08
5.	Number of fruits per cluster	64.32	13.22	22.46
6.	Fruit length	46.69	30.93	22.38
7.	Fruit width	80.23	5.07	14.69
8.	Fruit shape index	76.80	21.17	2.03
9.	Number of locules per fruit	90.22	7.02	2.76
10.	Average fruit weight	73.28	4.82	21.89
11.	Number of fruits per plant	39.41	5.81	54.78
12.	Yield per plant	64.18	8.32	27.51
13.	Yield per hectare	67.39	6.02	26.59
14.	Total soluble solids	33.82	25.04	41.14
15.	TYLCV incidence	21.95	39.90	38.15
16.	Bacterial wilt incidence	53.18	13.46	33.36

**Table 3:** Estimate of heritability and genetic advance of summer tomato.

Sl. No.	Characters	Broad Sense heritability (h^2^b)	Narrow Sense heritability (h^2^n)	Genetic advance (GA)
1.	Plant height at last harvest	70.59	0.01	0.69
2.	Number of branches per plant	36.83	0.002	0.008
3.	Days to 50% flowering	97.30	0.01	0.02
4.	Number of flowers per cluster	48.68	0.07	0.13
5.	Number of fruits per cluster	39.62	0.04	0.06
6.	Fruit length	76.67	0.02	0.03
7.	Fruit width	84.00	0.03	0.05
8.	Fruit shape index	79.72	0.0007	0.002
9.	Number of locules per fruit	89.27	0.13	0.27
10.	Average fruit weight	99.74	0.015	0.98
11.	Number of fruits per plant	97.78	0.02	0.52
12.	Yield per plant	99.72	0.034	0.062
13.	Yield per hectare	98.59	0.035	0.067
14.	Total soluble solids	99.03	0.02	0.03
15.	TYLCV incidence	79.80	0.02	0.19
16.	Bacterial wilt incidence	50.76	0.05	0.58

**Table 4a:** General combining ability effects of parents for different parameters in summer tomato.

Parents	Plant height at last harvest	No. of branches per plant	Days to 50% flowering	No. of flowers per cluster	No. of fruits per cluster	Fruit length	Fruit width	Fruit shape index
Lines								
AVTOV1001	15.33*	−0.01	1.07*	−0.08	−0.34	−0.22	0.18	−0.09*
AVTOV1002	−4.42	0.74	0.07	−0.16	−0.07	0.46**	0.51**	−0.02
AVTOV1006	5.53	−0.31	0.07	−0.51	−0.19	−0.12	0.37	−0.11**
AVTOV1007	1.88	−0.36	−1.43**	0.50	0.26	0.09	−0.49**	0.14**
AVTOV1008	−4.64	0.29	−0.43	0.31	0.21	−0.25	−0.34*	0.02
AVTOV1009	−14.87*	−1.21	1.57**	0.75**	0.58	−0.09	−0.34*	0.06
AVTOV1010	1.18	0.84	−0.93*	−0.81**	−0.44	0.13	0.11	0.00
S.E. (g_i_)	7.08	0.81	0.45	0.32	0.29	0.19	0.13	0.03
S.E. (g_i_ – g_j_) line	10.72	1.14	0.63	0.45	0.40	0.27	0.18	0.05
Testers								
C41	2.75	0.34	0.36	0.21	0.15	0.19	−0.01	0.04*
BARI-4	−2.75	−0.34	−0.36	−0.21	−0.15	−0.19	0.01	−0.04*
S.E. (g_t_) tester	4.05	0.43	0.24	0.17	0.15	0.10	0.07	0.02
S.E. (g_i_ – g_j_) tester	5.73	0.61	0.34	0.24	0.22	0.14	0.10	0.03

*, **: Indicates significance at 5 and 1% level of probability respectively, S.E. (g_i_) = Standard error (gca effect for lines), S.E. (g_i_ – g_j_) line = Standard error (between gca effect of two lines), S.E. (g_t_) = Standard error (gca effect for tester), S.E. (g_i_ – g_j_) = Standard error (between gca effect of two testers).

**Table 4b:** General combining ability effects of parents for different parameters in summer tomato.

Parents	No. of locules per fruit	Average fruit weight (g)	No. of fruits per plant	Yield per plant (kg)	Yield per hectare	Total soluble solids	TYLCV incidence	Bacterial wilt incidence
Lines								
AVTOV1001	0.74**	8.19**	0.78	−0.16**	1.89**	0.67**	0.14	1.40
AVTOV1002	0.52**	18.65**	4.08**	0.41**	1.28**	0.34*	3.82*	−0.70
AVTOV1006	1.22**	3.22**	2.18*	0.55**	2.98**	−0.03	−1.32	1.90
AVTOV1007	−0.68**	−12.22**	−10.50**	−0.88**	−4.26**	−0.12	−0.16	−2.40
AVTOV1008	−0.58**	−9.45**	7.43**	0.31**	0.85	−0.04	−1.41	−4.73*
AVTOV1009	−0.88**	−6.49**	−5.77**	−0.23**	−3.54**	−0.23	0.27	7.84**
AVTOV1010	−0.33	−1.90*	1.81	−0.01	0.80	−0.60**	−1.35	−3.32
S.E. (g_i_)	0.17	0.82	0.94	0.02	0.45	0.15	1.70	2.02
S.E. (g_i_ – g_j_) line	0.24	1.15	1.33	0.02	0.63	0.22	2.40	2.85
Testers								
C41	−0.21*	−2.58**	−2.17**	−0.16**	0.77**	0.33**	−2.29*	1.96
BARI-4	0.21*	2.58**	2.17**	0.16**	−0.77**	−0.33**	2.29*	−1.96
S.E. (g_t_) tester	0.09	0.44	0.50	0.01	0.24	0.08	0.91	1.08
S.E. (g_i_ –g_j_) tester	0.13	0.62	0.71	0.01	0.34	0.12	1.28	1.40

*, **: Indicates significance at 5 and 1% level of probability respectively, S.E. (g_i_) = Standard error (gca effect for lines), S.E. (g_i_ – g_j_) line = Standard error (between gca effect of two lines), S.E. (g_t_) = Standard error (gca effect for tester), S.E. (g_i_ – g_j_) = Standard error (between gca effect of two testers).

**Table 5a:** Specific combining ability effects of hybrids for different parameters in summer tomato.

Hybrids	Plant height at last harvest	No. of branches per plant	Days to 50% flowering	No. of flowers per cluster	No. of fruits per cluster	Fruit length	Fruit width	Fruit shape index
AVTOV1001 × C41	−18.90*	−0.49	−0.36	0.17	−0.03	−0.11	−0.10	−0.004
AVTOV1001 × BARI-4	18.90*	0.49	0.36	−0.17	0.03	0.11	0.10	0.004
AVTOV1002 × C41	−2.75	−0.34	−0.36	0.25	0.35	−0.25	−0.27	0.003
AVTOV1002 × BARI-4	2.75	0.34	0.36	−0.25	−0.35	0.25	0.27	−0.003
AVTOV1006 × C41	3.70	1.21	−0.36	0.20	0.17	0.01	−0.05	0.011
AVTOV1006 × BARI-4	−3.70	−1.21	0.36	−0.20	−0.17	−0.01	0.05	−0.011
AVTOV1007 × C41	3.85	−0.64	−0.86	−0.41	−0.13	0.28	0.22	0.013
AVTOV1007 × BARI-4	−3.85	0.64	0.86	0.41	0.13	−0.28	−0.22	−0.013
AVTOV1008 × C41	−5.13	−0.29	−0.86	−0.19	−0.23	−0.09	0.03	−0.022
AVTOV1008 × BARI-4	5.13	0.29	0.86	0.19	0.23	0.09	−0.03	0.022
AVTOV1009 × C41	18.50*	0.81	2.14**	0.10	0.10	0.08	0.14	−0.014
AVTOV1009 × BARI-4	−18.50*	−0.81	−2.14**	−0.10	−0.10	−0.08	−0.14	0.014
AVTOV1010 × C41	0.75	−0.24	0.64	−0.11	−0.23	0.07	0.03	0.013
AVTOV1010 × BARI-4	−0.75	0.24	−0.64	0.11	0.23	−0.07	−0.03	−0.013
S.E. (s_ij_)	8.72	1.14	0.63	0.45	0.40	0.27	0.18	0.046
S.E. (s_ij_- s_kl_) tester	15.16	1.61	0.89	0.63	0.57	0.38	0.25	0.066

*, **: Indicates significance at 5 and 1% level of probability respectively, S.E. (s_ij_) = Standard error (sca effects for crosses), S. E. (s_ij_- s_kl_) = Standard error (between sca effects of two crosses).

**Table 5b:** Specific combining ability effects of hybrids for different parameters in summer tomato.

Hybrids	No. of locules per fruit	Average fruit weight (g)	No. of fruits per plant	Yield per plant (kg)	Yield per hectare	Total soluble solids	TYLCV incidence	Bacterial wilt incidence
AVTOV1001 × C41	−0.21	2.08	−4.18**	−0.13**	−1.66*	−0.31	1.11	0.14
AVTOV1001 × BARI-4	0.21	−2.08	4.18**	0.13**	1.66*	0.31	−1.11	−0.14
AVTOV1002 × C41	0.21	−10.02**	−6.28**	0.13**	0.89	0.53*	−5.21*	0.66
AVTOV1002 × BARI-4	−0.21	10.02**	6.28**	−0.13**	−0.89	−0.53*	5.21*	−0.66
AVTOV1006 × C41	0.01	1.76	2.22	0.45**	1.00	−0.41	−0.06	3.58
AVTOV1006 × BARI-4	−0.01	−1.76	−2.22	−0.45**	−1.00	0.41	0.06	−3.58
AVTOV1007 × C41	0.11	4.15**	−5.25**	−0.26**	−2.05**	−0.25	1.27	−2.65
AVTOV1007 × BARI-4	−0.11	−4.15**	5.25**	0.26**	2.05**	0.25	−1.27	2.65
AVTOV1008 × C41	0.01	3.03**	6.87**	0.38**	1.98	−0.46*	2.06	1.50
AVTOV1008 × BARI-4	−0.01	−3.03**	−6.87**	−0.38**	−1.98	0.46*	−2.07	−1.50
AVTOV1009 × C41	0.01	5.72**	−5.43**	−0.22**	1.58*	0.40	0.98	−6.04*
AVTOV1009 × BARI-4	−0.01	−5.72**	5.43**	0.22**	−1.58*	−0.40	−0.98	6.04*
AVTOV1010 × C41	−0.14	−6.72**	12.05**	−0.35**	−1.75*	0.49*	−0.15	2.80
AVTOV1010 × BARI-4	0.14	6.72**	−12.05**	0.35**	1.75*	−0.49*	0.15	−2.80
S.E. (s_ij_)	0.24	1.15	1.33	0.02	0.63	0.22	2.40	2.85
S.E. (s_ij_- s_kl_) tester	0.34	1.63	1.88	0.03	0.89	0.31	3.39	4.04

*, **: Indicates significance at 5 and 1% level of probability respectively, S.E. (s_ij_) = Standard error (sca effects for crosses), S. E. (s_ij_- s_kl_) = Standard error (between sca effects of two crosses).

## 4 Experimental Design, Materials and Methods

### 4.1 Description of experimental site

The research work was conducted at the Olericulture division of the Horticulture Research Centre (HRC) of Bangladesh Agricultural Research Institute (BARI), Gazipur. The experimental area was situated at 23°59′27.7"N latitude and 90°24′42.4"Elongitude at an altitude of 8.4 meters above sea level. The experimental field belongs to the Agroecological zone of “The Modhupur Tract” (AEZ-28). This is a region of complex relief and soils developed over the Modhupur clay, where floodplain sediments buried the dissected edges of the Modhupur Tract leaving small hillocks of red soils as ‘islands’ surrounded by floodplain. The climate of the experimental site is subtropical characterized by heavy rainfall from May to October and scanty during the rest of the year (Supplementary Table 1).

### 4.2 Plant material used in this study

The study consisted of seven inbred lines as female parent (L) and two testers as male parent (T) with diverse genetic bases and heat tolerance quality. Fourteen cross combinations were produced through L × T mating design. Seven female parents include AVTOV1001, AVTOV1002, AVTOV1006, AVTOV1007, AVOV1008, AVTOV1009 and AVTOV-1010. Two male parental lines were C41 and BARI-4. Seeds of the line, tester and their hybrids were sown in the well-prepared seedbed. Forty-day-old seedlings were transplanted in the main field in the same location where F_1_ s (experimental hybrids) were synthesized.

### 4.3 Synthesis of F_1_ s populations

The parental seeds were sown during winter in the nursery bed. Four-week-old seedlings were transplanted in crossing blocks at the spacing of 60 cm × 60 cm. Healthy flower buds in a cyme preferably of the first flush which were expected to open in the next day were selected for emasculation and pollination. The selected buds were emasculated by hand using forceps in the evening between 4.00 pm to 6.00 pm. The emasculated flowers (1 to 2 per cyme) were covered with red-coloured butter paper bags to avoid contamination. The pollination of emasculated flowers was done the next day morning from 7.00 am to 10.30 am. Well-opened flowers with dehisced anthers were collected from the male parents, the butter paper bag was removed carefully and the stigma was brought in contact with dehisced anthers of male flowers. The female flower was covered with a white-coloured butter paper bag immediately after pollination for easy identification and to further avoid contamination by foreign pollen. The pedicel of each pollinated flower was tied with a label bearing information about female and male parents and the date of crossing for identification. The ripe crossed fruits and selfed fruits of parents were harvested. Seeds were extracted from red ripe fruits by fermentation method. Extracted seeds were dried properly. Dried seeds were labelled and stored at a temperature of 8°C and relative humidity of 40%.

### 4.4 Design and layout of the experiment

The experiment was laid out in Randomized Complete Block Design (RCBD) with two replications. Seeds of the line, tester and their hybrids were sown in the well-prepared seedbed on 18th May 2012 and then forty-day-old tomato seedlings were transplanted in the main field under transparent polytunnels. The polytunnels were 2.3 m wide having two-unit beds with 0.8 m × 1 m sizes keeping a 30 cm drain in between 14-unit beds. Each unit bed contained double rows accommodating 24 plants.

### 4.5 Management practices and intercultural operations

The land was manured and fertilized at cow-dung 10 t/ha, urea 550 t/ha, TSP 450 kg/ha, MOP 250 kg/ha and gypsum 100 kg/ha. The 50% amount of cow-dung was applied at the final land preparation. The remaining half of cow-dung, entire amount of TSP and one third of urea and MOP were applied at the time of pit preparation and the rest were applied in three equal splits at 20, 40 and 60 days after planting (DAP) and mixed thoroughly with the soil. Intercultural operations such as weeding, mulching, irrigation etc. were done when necessary for proper growth and development of the plants. Proper shading was given in the morning at the first stage of transplanting to protect the young seedlings from scorching sunshine during the daytime. The population in each unit plot was sprayed with Tomatotone hormone (75 mg of 4-chlorophenoxy acetic acid per litre of distilled water) three times during flowering and fruit stages [9]. During the early stage of growth pruning was done by removing lateral branches below the first inflorescence to expose the plants to more sunlight. Staking was done with the bamboo sticks.

### 4.6 Observations and collection of data

Observations of all the 16 characters were recorded for each of line, tester and their hybrid. The morphological traits of summer tomato were recorded following the guidelines outlined in the International Plant Genetic Resources Institute (IPGRI) Descriptor for Tomato (*Lycopersicon* spp.) [10] . Fruits per plant, yield per plant and yield per hectare were calculated from the plot yield. Instructions for collecting data on various traits are outlined below.

#### 4.6.1 Plant height at last harvest (cm)

The height of the plants was measured from the base to the tip of the five randomly selected tagged plants in centimetres (cm) and the average was calculated.

#### 4.6.2 Number of branches per plant

The number of primary branches per plant was counted at the final harvest from 5 tagged plants and the average was calculated.

#### 4.6.3 Days to 50 per cent flowering

The number of days from transplanting to the first flower presence in 50 per cent of the plants in each row was recorded and the average was calculated.

#### 4.6.4 Number of flowers per cluster

Flowers of each cluster starting from 1 to 5 clusters were counted from each of 5 selected plants and their average was taken as the number of flowers per cluster.

#### 4.6.5 Number of fruits per cluster

Before first picking, three fruit bunches were chosen at random in each of the labelled plants to calculate the average number of fruits per cluster.

#### 4.6.6 Fruit length (Polar diameter, L)

The length of the fruit was measured in centimeters (cm) from the base of the calyx to the tip of the fruit with the help of vernier calipers, with a random sample of 10 fruits per plot.

#### 4.6.7 Fruit width (Equatorial diameter, D)

The diameter of the fruit was measured in centimeters (cm) with the help of vernier calipers at the center (equatorial length) of the fruit, with a random sample of 10 fruits per plot.

#### 4.6.8 Fruit shape index

The polar (L) and equatorial diameters (D) of the fruits were measured using digital Vernier calipers, and the fruit shape index was calculated as the ratio of L/D.

#### 4.6.9 Number of locules per fruit

The number of locules was counted by cutting the fruit transversely in the middle and calculating the average.

#### 4.6.10 Average fruit weight (g)

Well-developed individual fruit weight was recorded in grams by weighing on a sensitive balance, with a random sample of 10 fruits per plot.

#### 4.6.11 Number of fruits per plant

The total number of fruits harvested from all the pickings was pooled and the average number of fruits was calculated across all the plots.

#### 4.6.12 Yield per plant (Kg)

Fruit yield was determined by adding the total fruit weight over all the pickings from each reference plant and expressed in kilograms (kg).

#### 4.6.13 Yield per hectare

Yield per hectare was determined by adding the total fruit weight over all the pickings from each plot of a parent/hybrid and converting to tones per hectare (t/ha).

#### 4.6.14 Total soluble solids (TSS)

A drop of juice was used to measure the Total Soluble Solids (TSS%) at ambient temperature using an Erma hand refractometer.

#### 4.6.15 Tomato yellow leaf curl virus (TYLCV) incidence

Observations were recorded at 50 days after transplanting. The per cent disease incidence was calculated using the formula below.

$$Per\;cent\;disease\;incidence\;=\;\frac{\text{Number}\;\text{of}\;\text{infected}\;\text{plants}\;\text{in}\;\text{the}\;\text{plot}}{\text{Total}\;\text{number}\;\text{of}\;\text{plants}\;\text{in}\;\text{the}\;\text{plot}}\times \;100$$

#### 4.6.16 Bacterial wilt disease incidence

The number of plants wilted in each plot in the field was recorded and expressed as per cent. They were scored as follows flowing the scale given by Mew and Ho [11] (Table 6).

**Table 6:** Classification of bacterial wilt severity based on percentage Incidence.

Reaction	Per cent wilt
Resistant	< 20%
Moderately resistant	20–40%
Moderately susceptible	40–60%
Susceptible	> 60%

### 4.7 Statistical analysis

The line × Tester (L × T) mating design is an extension of top cross analysis in the sense that instead of tester, as used in top crossing, more than one tester was employed under the L × T mating design [12]. The data analysis was conducted on the “agricolae” package of R statistical analysis software (Version 3.0.1) following the Line × Tester analysis method proposed by Kempthorne [12,13].

#### 4.7.1 Analysis of variance

Analysis of variance (ANOVA) for individual character was carried out based on mean value per treatment per replication following the procedure described by Panse and Sukhatme [14]. Analysis was carried out only to know the significance or otherwise of the difference seen between means of parents and hybrids. The significance of treatments was tested at 5 and 1 per cent probability. The model of analysis of variance table adopted is given below- (Table 7)

**Table 7:** The skeleton of the L × T ANOVA for parents and hybrids.

Source of variation	Degrees of freedom	Mean sum of squares
Replication	(r-1)	
Treatments	(e-1)	
Parents	(p-1)	
Parents Vs Crosses	1	
Crosses	(lt-1)	
Lines	(l-1)	M_1_
Testers	(t-1)	M_2_
Line × Tester	1	M_3_
Error	(e-1) (r-1)	M_4_
Total	(ltr-1)	

Where,

r = Number of replications.

e = Number of treatments.

l = Number of lines.

t = Number of testers.

#### 4.7.2 Combining ability analysis

##### 4.7.2.1 Analysis of variance for combining ability

For combining ability analysis, only crosses were considered and analyzed based on the line × tester analysis as proposed by Kempthorne [12] and emphasized by Arunachalam [15]. Analysis of variance adopted for combining ability is given below-

The analysis was based on the following mathematical model-

(1) $$Y_{ij}K=\mu +g_{i}+g_{j}+s_{ij}+e_{ij}k$$

Where,

Yijk = Any character measured of the cross (i × j) in the *k*th replication.

µ = Population mean

g_i_ = Gca effect of *i*th parent

g_j_ = Gca effect of *j*th parent

S_ij_ = Sca effect of (i × j)th cross

e_ijk_ = Random errors effect associated with (i × j)th observation in *k*th replication

i = Number of female parents

j = Number of male parents

k = Number of replications

COV (half-sibs) and COV (full-sibs) were estimated by equating the observed mean squares to their expectations. Since the number of lines and testers used were different, the weighted average of COV (half-sibs) was computed by deriving least square estimates as proposed by Arunachalam [15].

The least-square estimates were derived as follows-

(2) $$Y\;=\;COV\;\left(half-sibs\right)\;=\;\frac{Y2\;[t(a+c-2b)\;+\;d\;(b+c-2a)}{\text{dt}-t^{2}-d^{2}}$$

(3) $$X\;=\;COV\;\left(full\;sibs\right)\;=\;\frac{\left[t\left(a+c-2b\right)+\;d\;\left(b+c-2a\right)-\frac{1}{2}\left[t^{2}\left(a+c\right)+d^{2}\left(b+c\right)-d+\left(a+b\right)\right]\right]}{\text{dt}-t^{2}-d^{2}}$$

Where,

$$a\;=\;\left(M_{2}-M_{4}\right)/r,\;b=\;\left(M_{1}-M_{4}\right)/r,\;c=\;\left(M_{3}-M_{4}\right)/r$$

The least-square estimates of X and Y were used to compute components of combining ability variances as follows (Table 8).

s²gca=Yands²gca=X−2Y

**Table 8:** ANOVA for combining ability.

Source	d.f	MSS	Expectation
Replication	(r-1)	–	
Crosses	(dt-1)	–	
Lines	(d-1)	M_1_	σ²_e_ + rσ²_d_ + trσ²_1_
Testers	(t-1)	M_2_	σ²_e_ + rσ²_d_ + drσ²_t_
Lines × Testers	(d-1) (t-1)	M_3_	σ²_e_ + rσ²_d_
Error (L × T)	(r-1) (dt-1)	M_4_	σ²_e_

Where,

d = number of lines.

t = number of testers.

##### 4.7.2.2 Estimation of combining ability effects

The combining ability effects were estimated as follows:

General combining ability effects (gca effects) of *i*th line

(4) $$g_{i}=\;\frac{\text{xi}.\;.}{\text{tr}}\;-\;\frac{x\;.\;.\;.}{\text{dtr}}$$

Where,

xi. . = Total of the *i*th line overall testers and replication and

x. . . /dtr = Overall mean

General combining ability effects (gca effects) of *j*th tester

(5) $$g_{i}=\frac{x.j.\;.}{\text{dr}}-\;\frac{\;x\;.\;.\;.}{\text{dtr}}$$

Where,

x.j. = Total of *j*th tester over all the lines and replications

Specific combining ability effects (sca) due to *ij*th cross

(6) $$S_{ij}\;=\;\frac{\text{xij}\;.}{r}\;-\;\frac{x\;.\;.\;.}{\text{dtr}}-\;\frac{x.j.}{\text{dr}}-\;\frac{x\;.\;.\;.}{\text{dtr}}$$

Where,

x_ij_. = Total of *ij*th cross over all the lines and replications

##### 4.7.2.3 Standard errors of combining ability estimates

The variance of different estimates was calculated by multiplying the error variance by their respective coefficients as shown below-

Error variance = M_4_ = Error mean squares from combining ability analysis

Variance of (g_i_) lines = $\frac{M4}{\text{rt}}$

Variance of (g_j_) lines = $\frac{M_{4}}{\text{rd}}$

Variance of hybrids (S_ij_) = $\frac{M_{4}}{r}$

The square root of the variance of the estimates was used as a standard error meant for testing the significance of combining ability effects.

#### 4.7.3 Heritability analysis

The narrow sense heritability formula was estimated based on the assumption that the total variation among hybrids equals twice the GCA variance plus SCA variance while the genetic variance corresponds to the variance of additive effects [16,17].

(7) $$h^{2}\;\frac{\sigma^{2}A}{\sigma^{2}P}\;=\;\frac{\sigma^{2}A}{\sigma^{2}A+\sigma^{2}D}\;=\;\frac{2\sigma^{2}\text{GCA}}{2\sigma^{2\;}\text{GCA}+\;\sigma^{2\;}\text{SCA}}$$

In which, the additive variance: σ^2^ A = 2σ^2^ GCA; the dominant variance: σ^2^ D = σ^2^ SCA

#### 4.7.4 Proportion contribution of lines, testers and their interaction

The percent contribution of lines, testers, and their interaction to the total variance in each character was estimated following the method of Singh and Chaudhary [18] as given below.

(8) $$Contribution\;of\;lines\;=\;\frac{\text{SS}(d)}{\text{SS}(c)}\;\times \;100$$

Where,

SS(d) = Sum of squares due to line effect

SS(c) = Sum of squares due to crosses

(9) $$Contribution\;of\;testers\;=\;\frac{\text{SS}\left(t\right)}{\text{SS}\left(c\right)}\;\times \;100$$

Where,

SS(t) = Sum of squares due to testers effect

(10) $$Contribution\;of\;\left(line\;\times \;tester\right)\;=\;\frac{\text{SS}\;(\text{dxt})}{\text{SS}\;(c)}\;\times \;100$$

Where,

SS (dxt) = Sum of squares due to interaction

## Limitations

None.

## Ethics Statement

All authors have read and followed the ethical requirements for publication in Data in Brief and our work meets these requirements. Our work does not involve studies with animals and humans.

## CRediT author statement

**Mohammad Matin Akand**: Conceptualization, Methodology, Software, Validation, Formal analysis, Investigation, Visualization, Writing – original draft, Writing –review & editing, **Mohammed Abu Taher Masud**: Supervision, Writing - Review & Editing, Data curation, **Md. Azizul Hoque**: Supervision, Writing - Review & Editing, **Mohammad Mostafa Kamal**: Data curation, Writing - Review & Editing, **Mohammad Rezaul Karim**: Writing - Review & Editing, **Bahauddin Ahmed:** Data curation.

## Data Availability

Mendeley DataData set for estimating combining abilities for yield and quality attributes in summer tomato using line by tester analysis in Bangladesh (Original data).

## Appendix Supplementary materials

Image, application 1
