# Supplementary material for: Data set for estimating combining abilities for yield and quality attributes in summer tomato using line by tester analysis in Bangladesh
Source: Data Brief. 2024 Oct 31;57:111063. doi: 10.1016/j.dib.2024.111063 (PMC11585899; doi:10.1016/j.dib.2024.111063)
Supplement: Supplementary file 1 [file mmc1.docx]

|  |  |
| --- | --- |
| 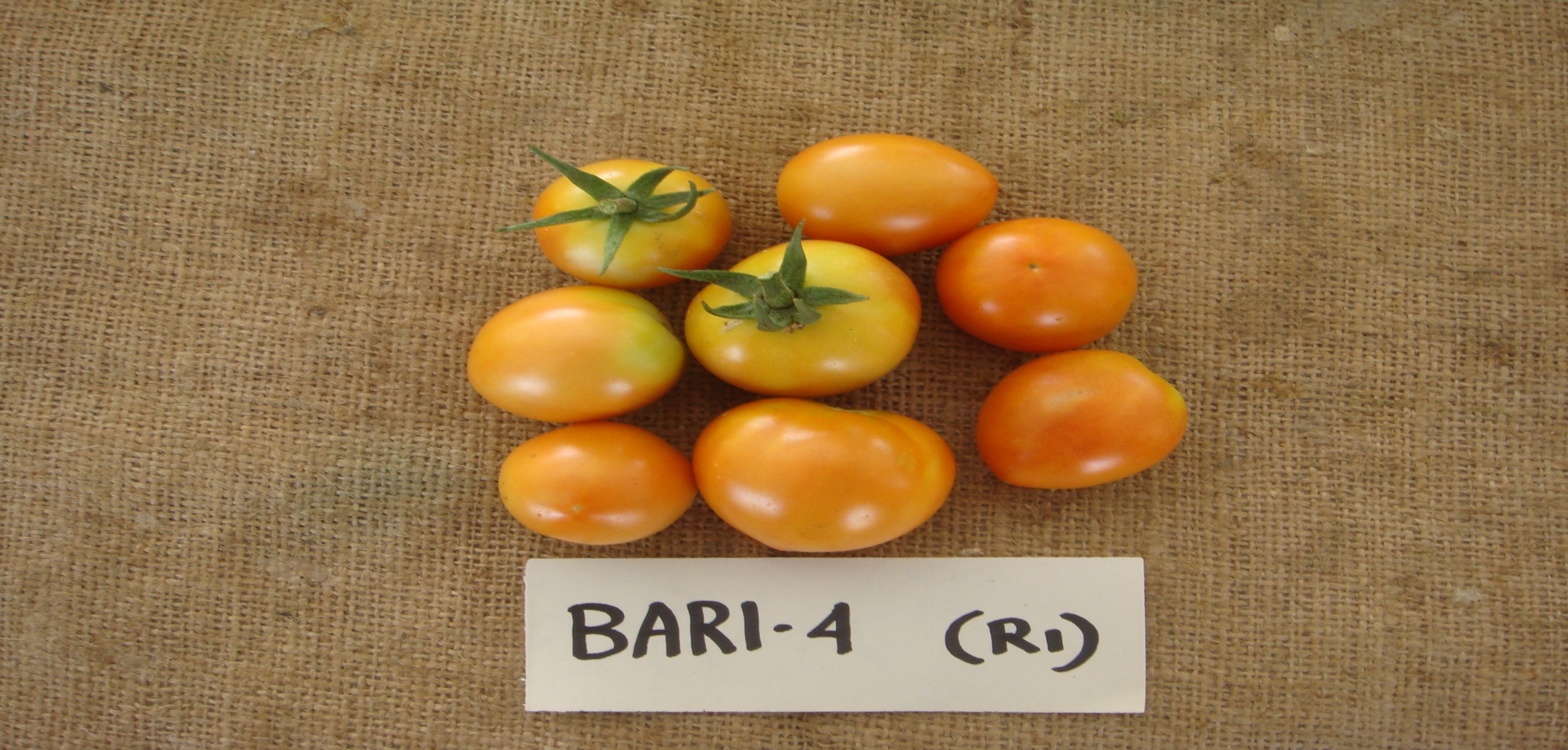 **BARI-4** 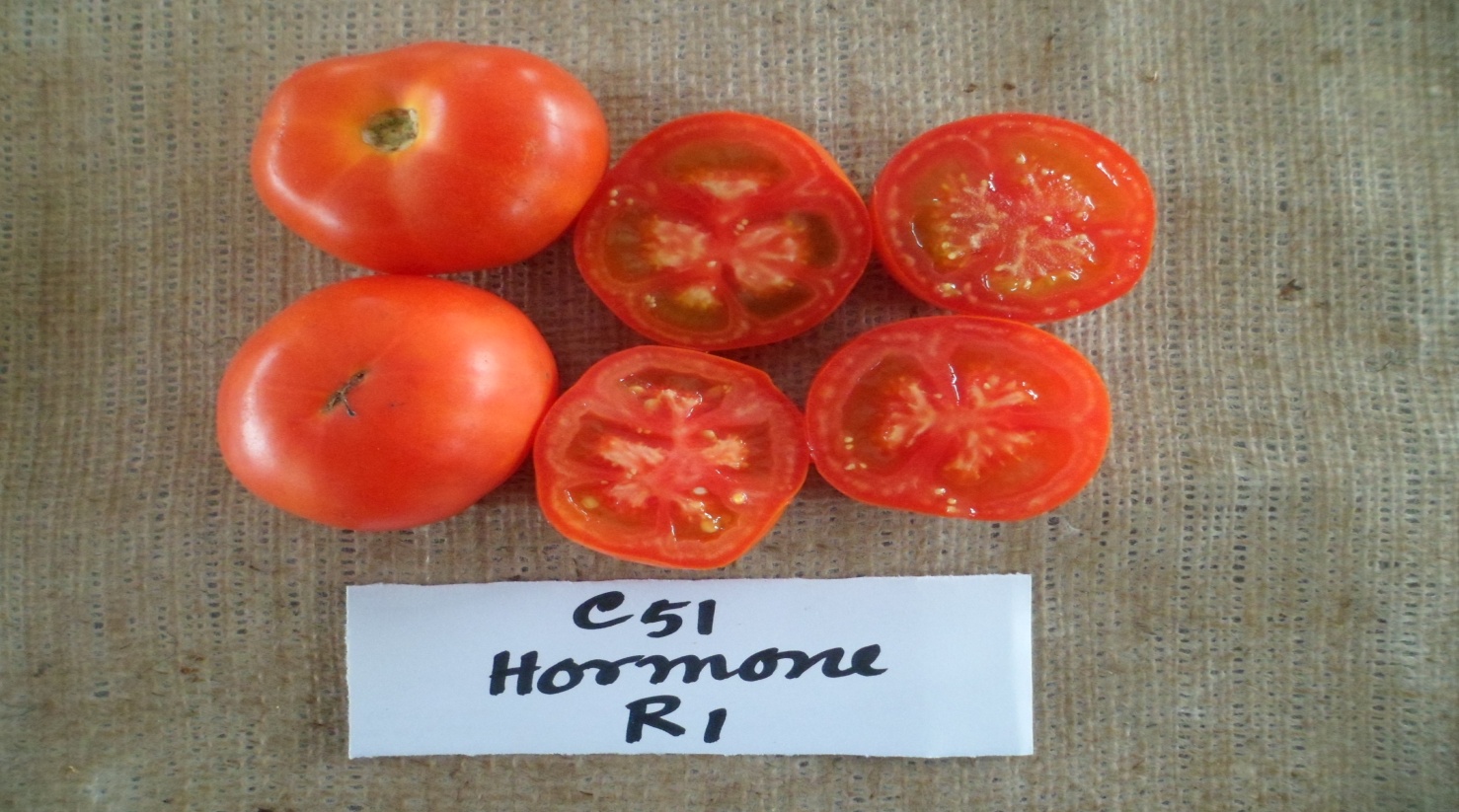 **C41**  Fig.2 Fruits of the tester of summer tomato involved in crossing 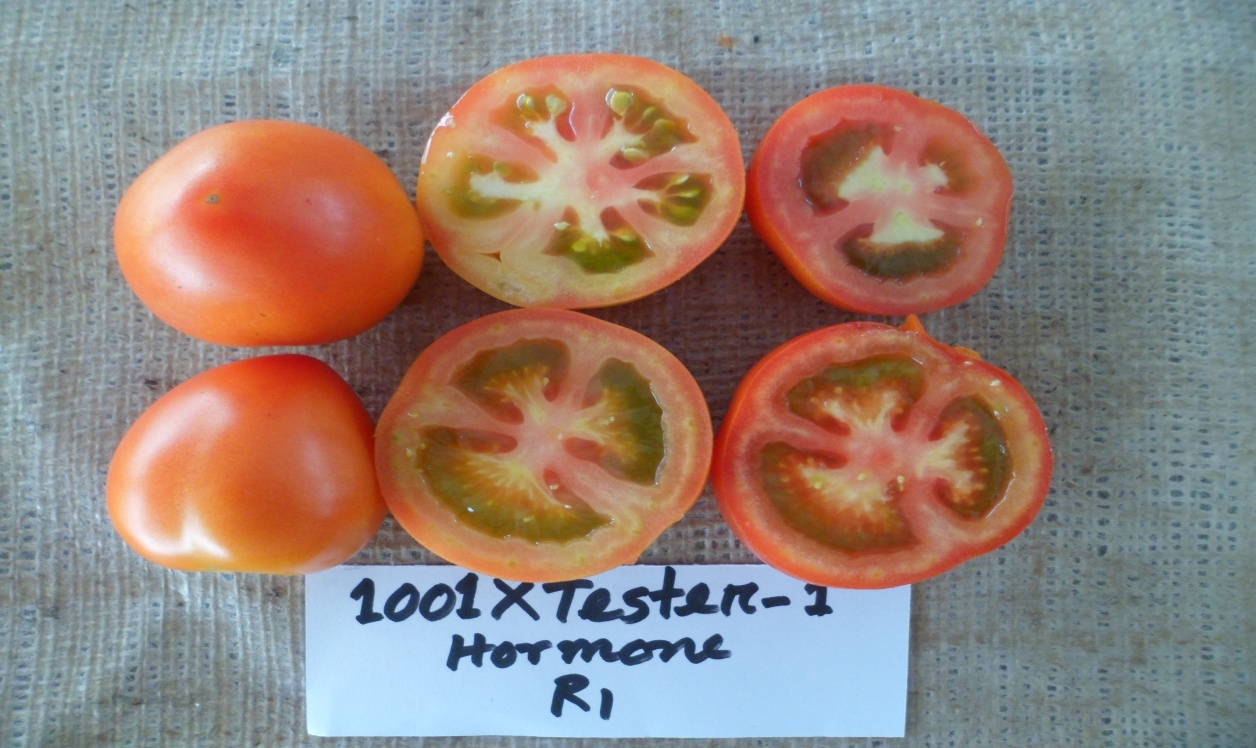 **AVTOV1001 × C41** 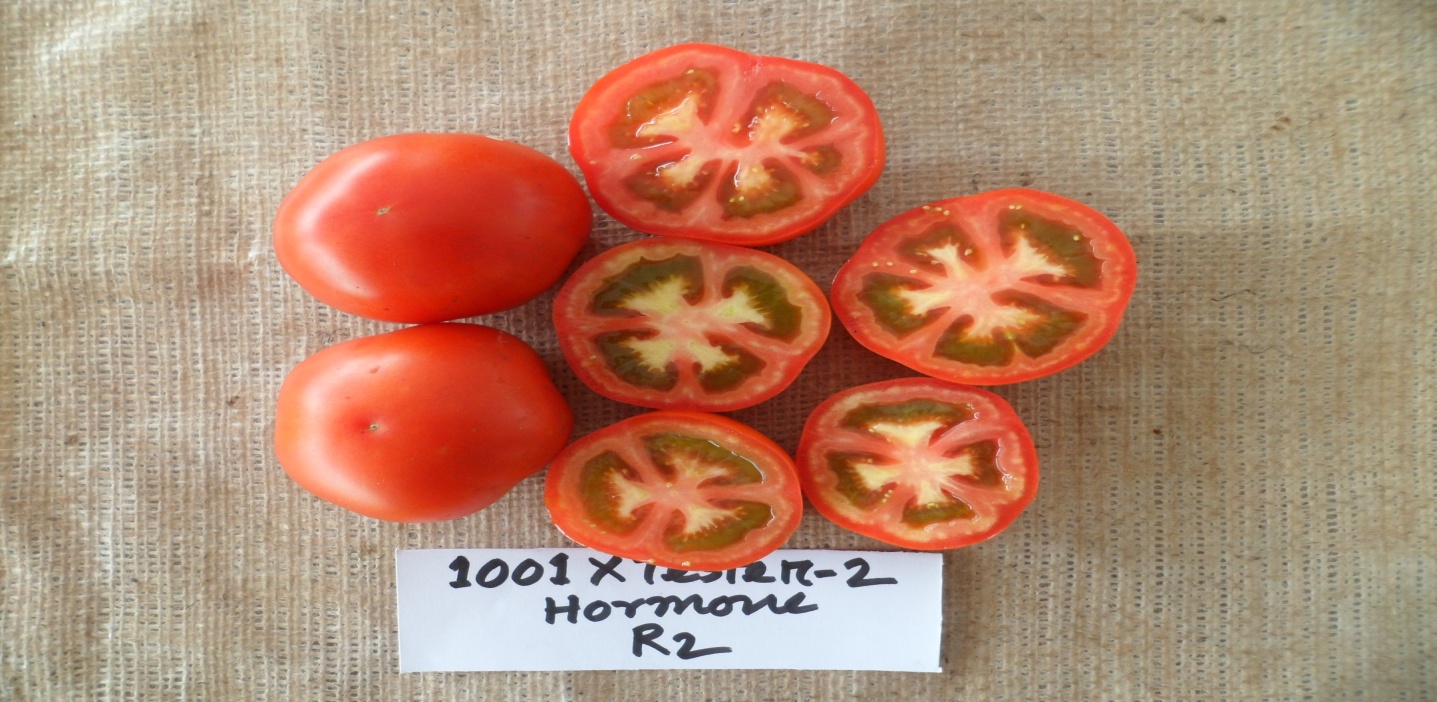 **AVTOV1001 × BARI-4** 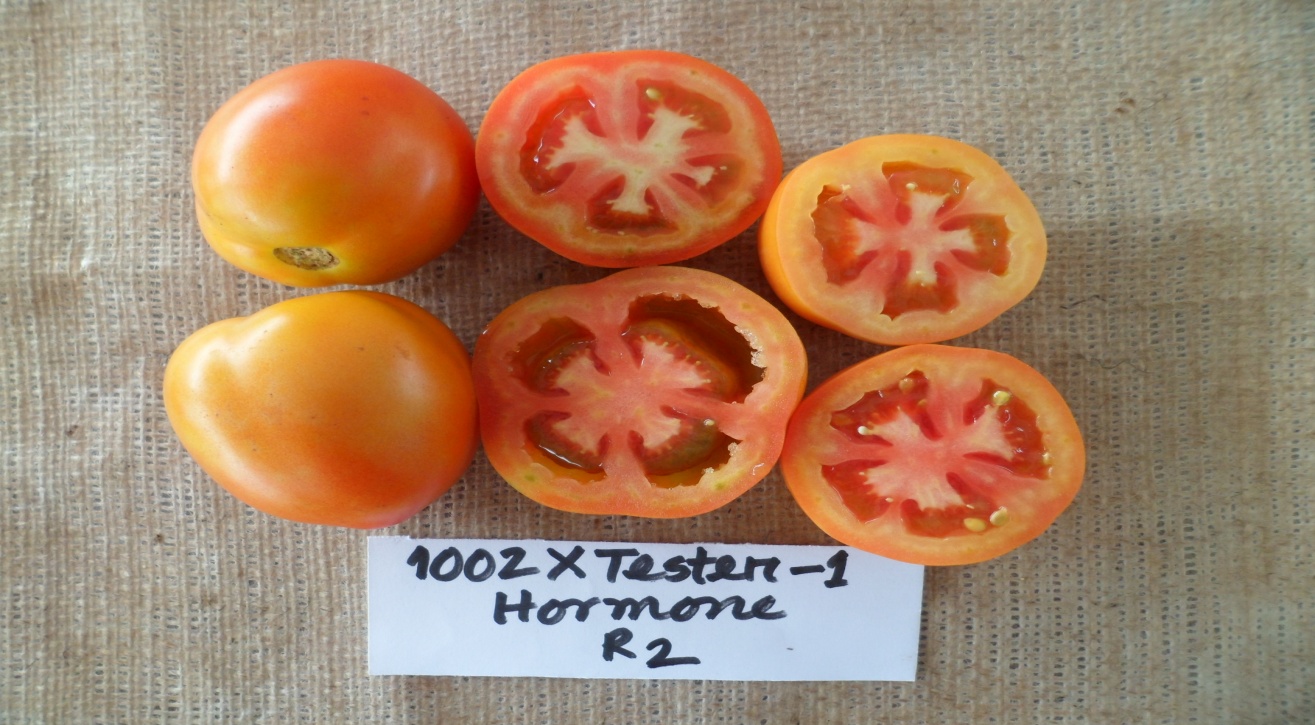 **AVTOV1002 × C41** 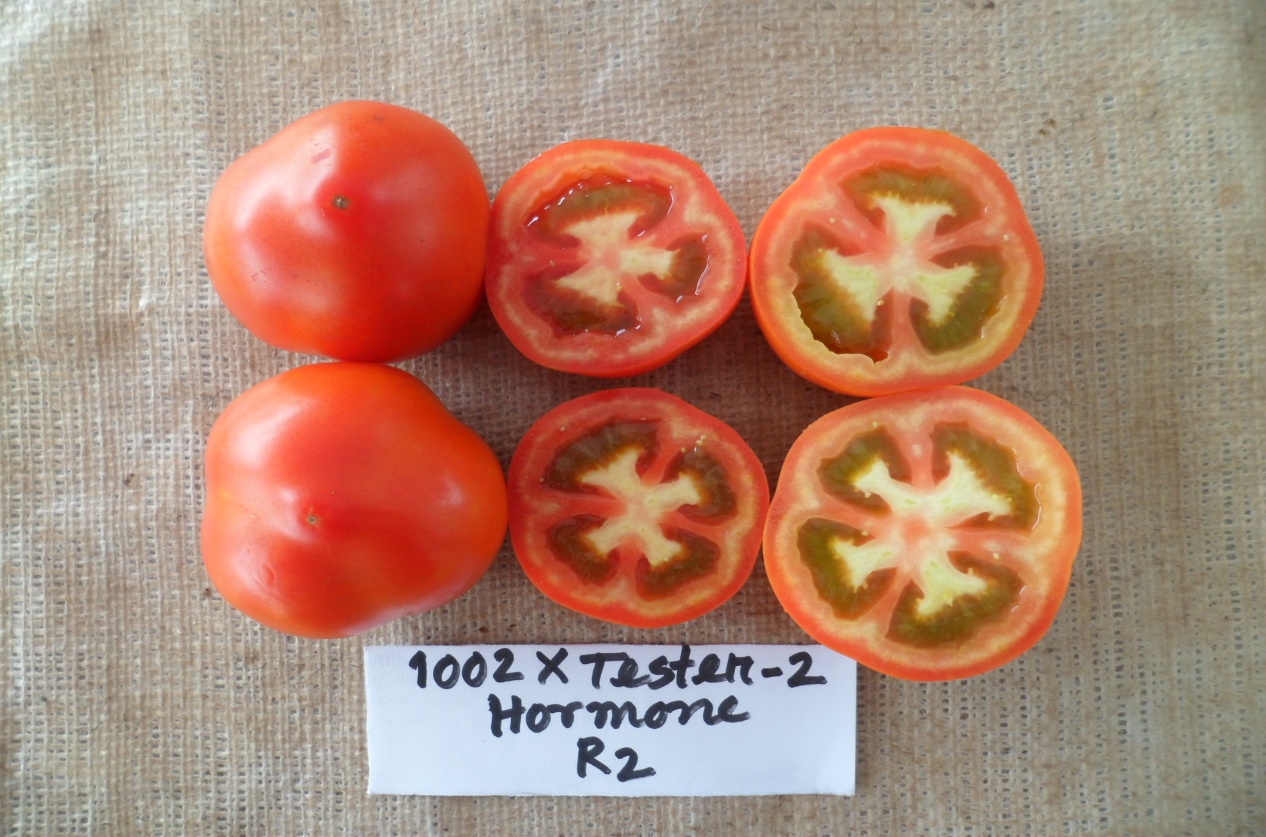 **AVTOV1002 × BARI-4** 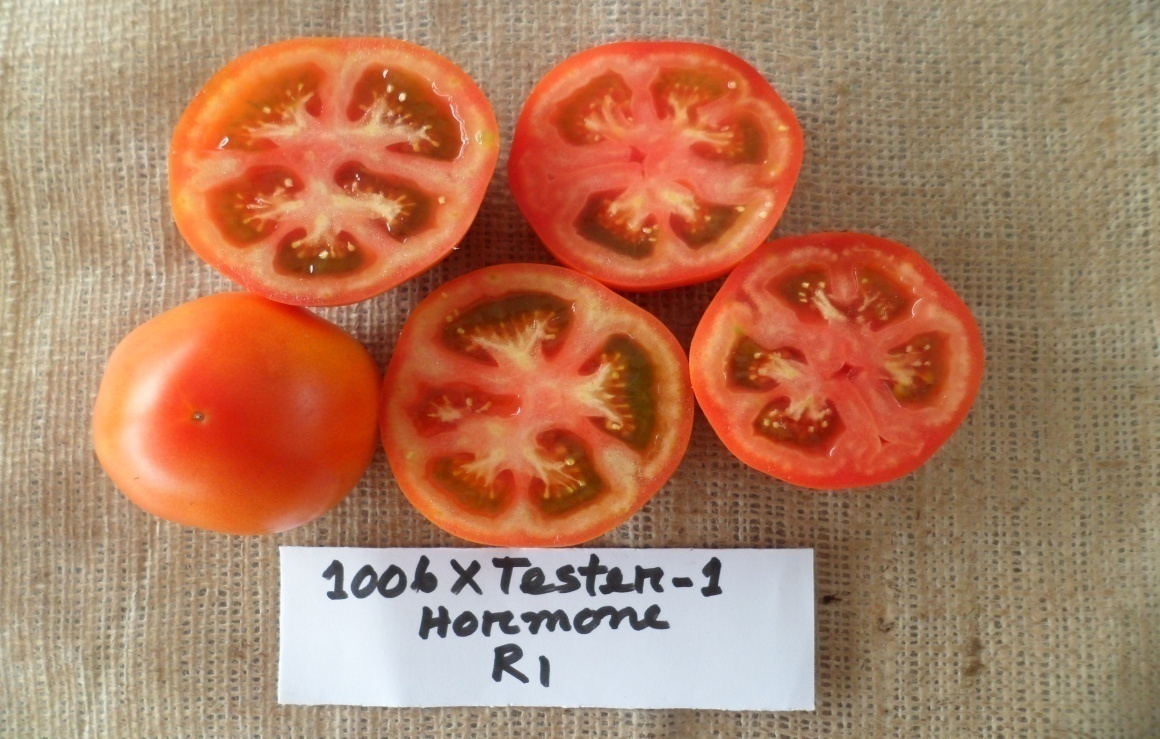 **AVTOV1006 × C41** 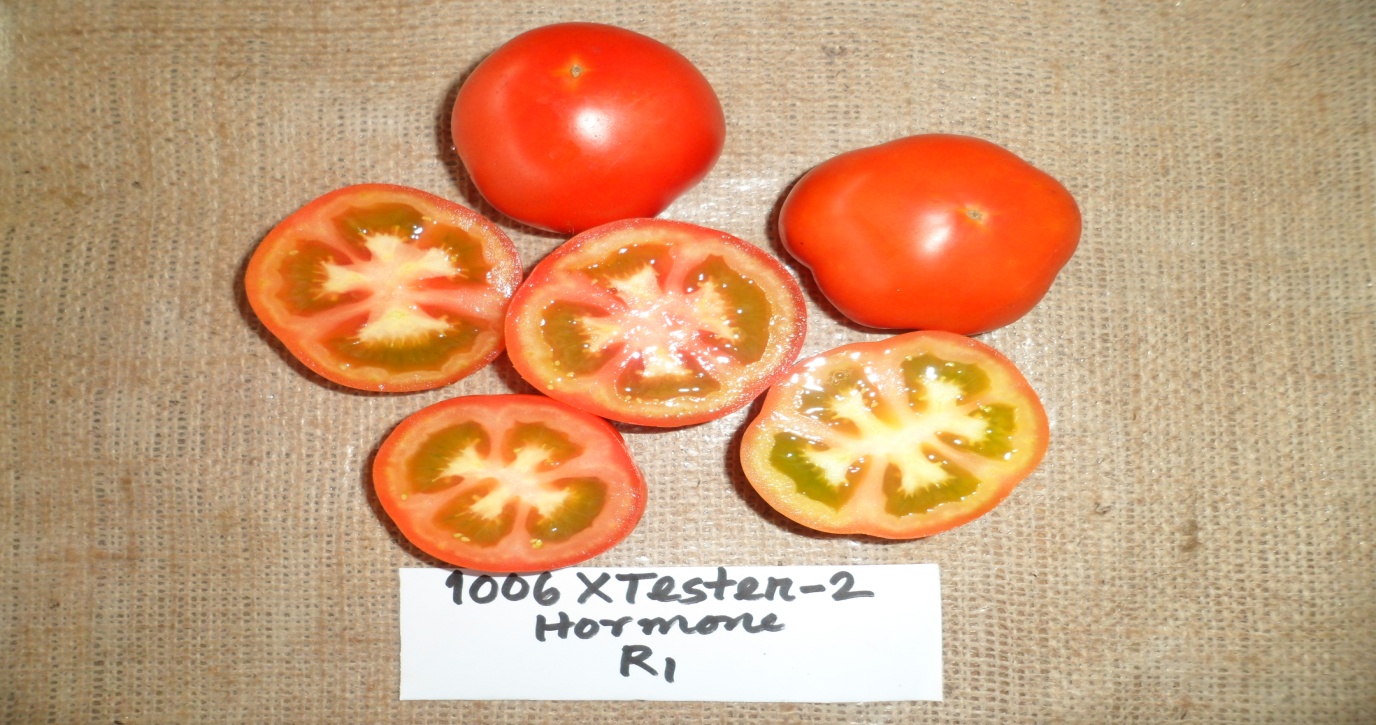 **AVTOV1006 × BARI-4** |  |
|  |  |
|  |  |

Fig. 3(a). Fruits of summer tomato hybrids

| 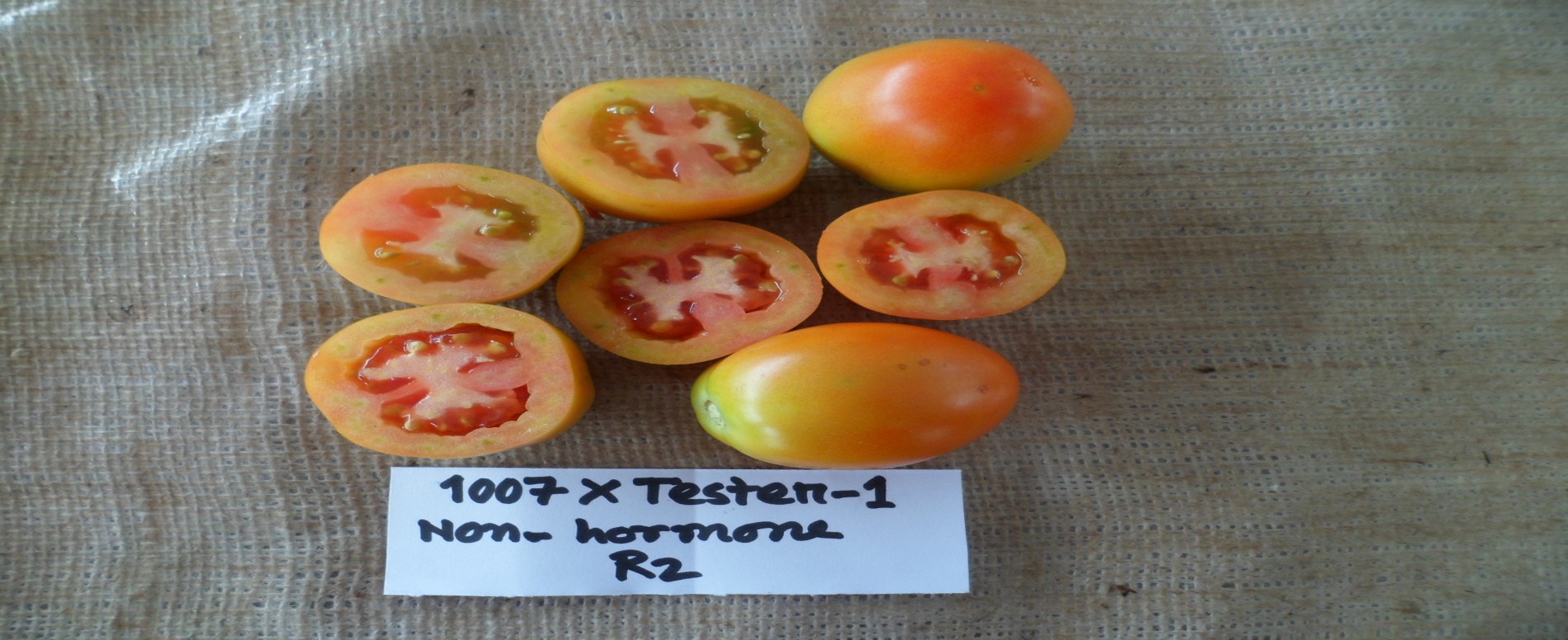 **AVTOV1007 × C41** 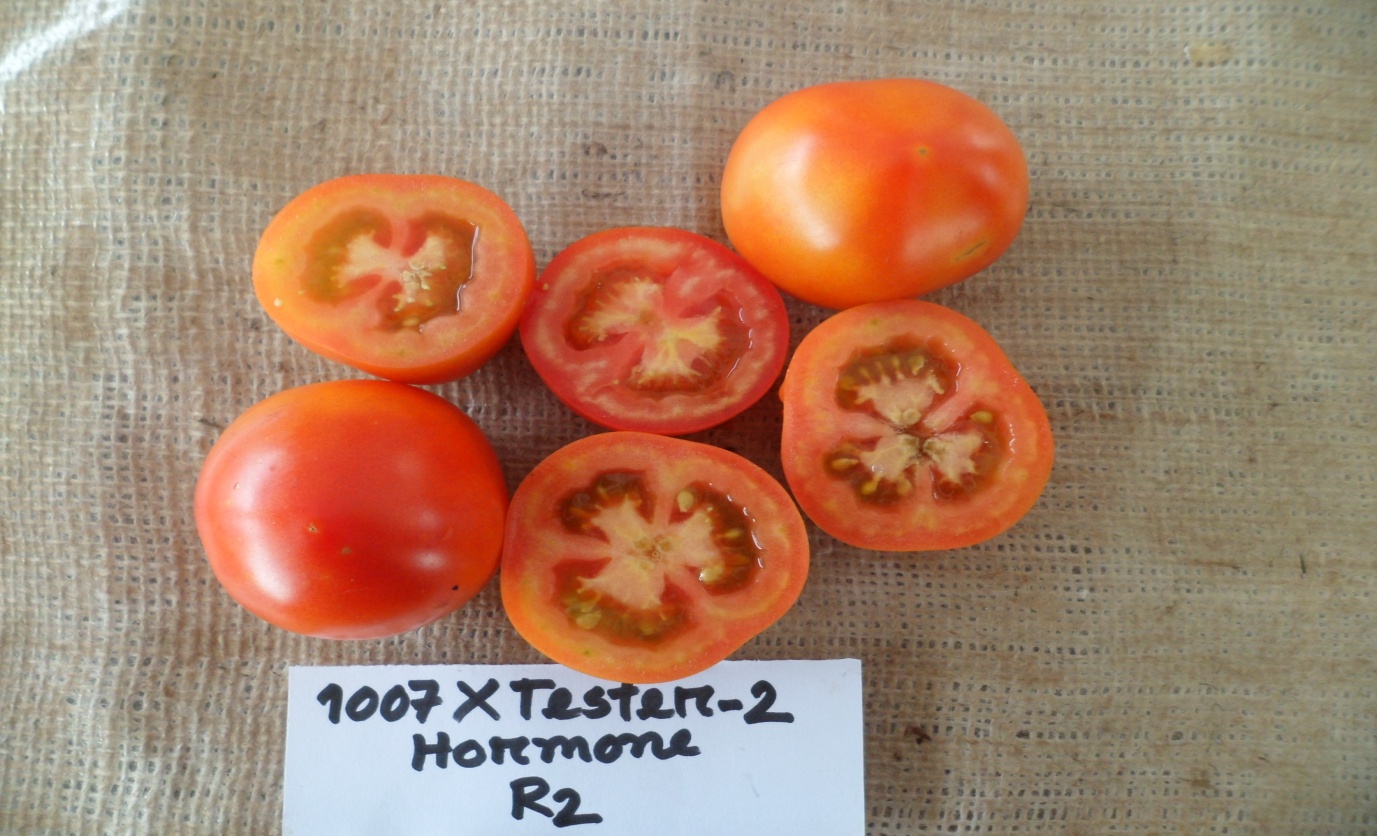 **AVTOV1007 × BARI-4** 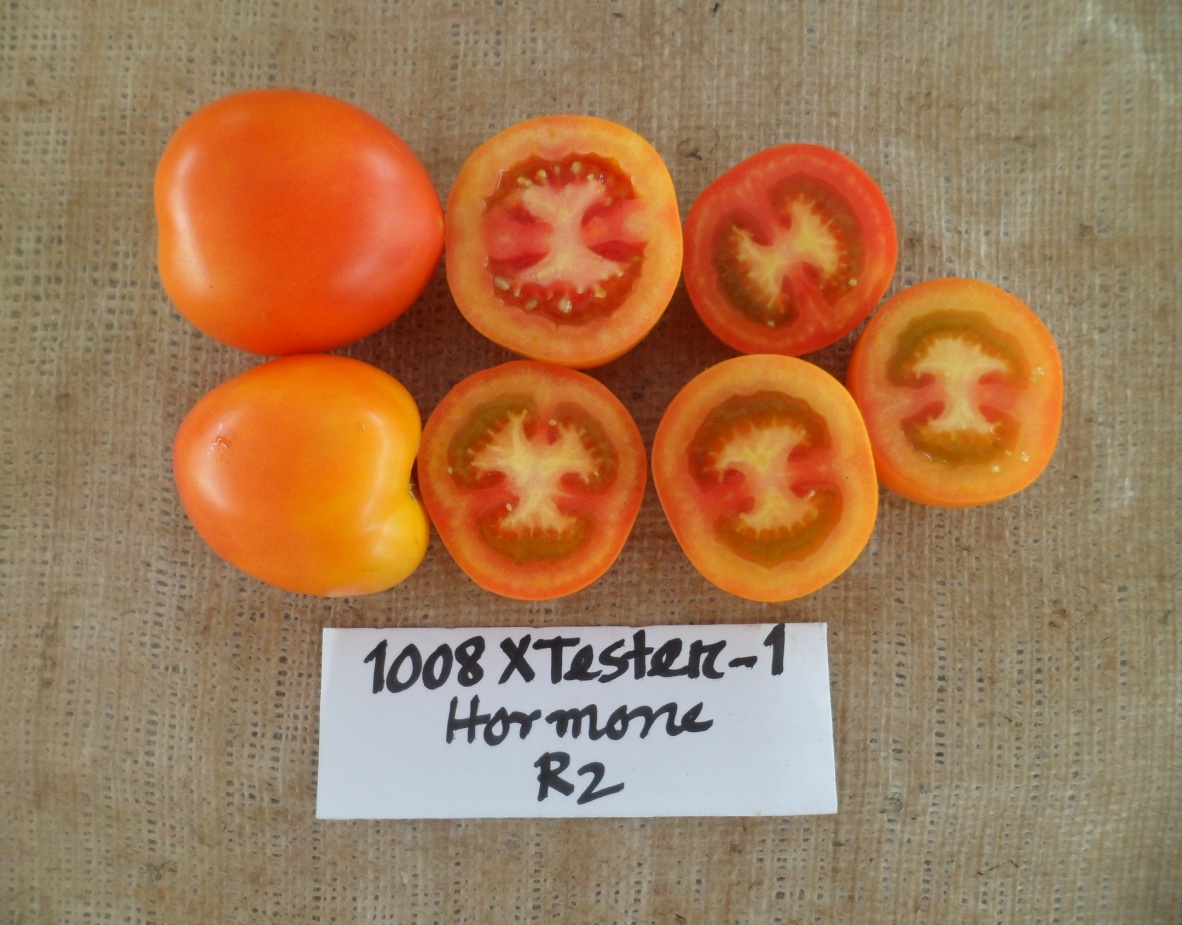 **AVTOV1008 × C41** 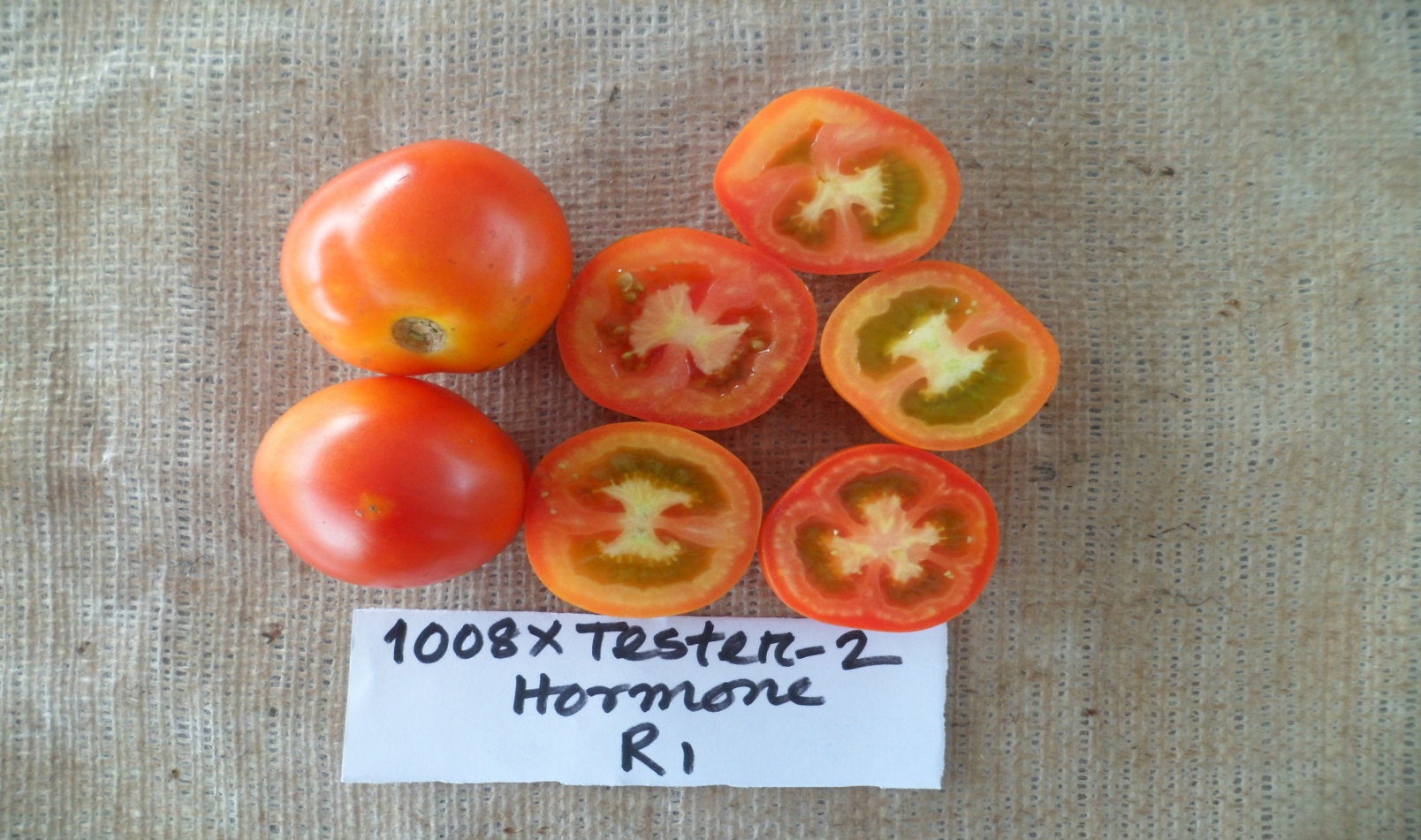 **AVTOV1008 × BARI-4** 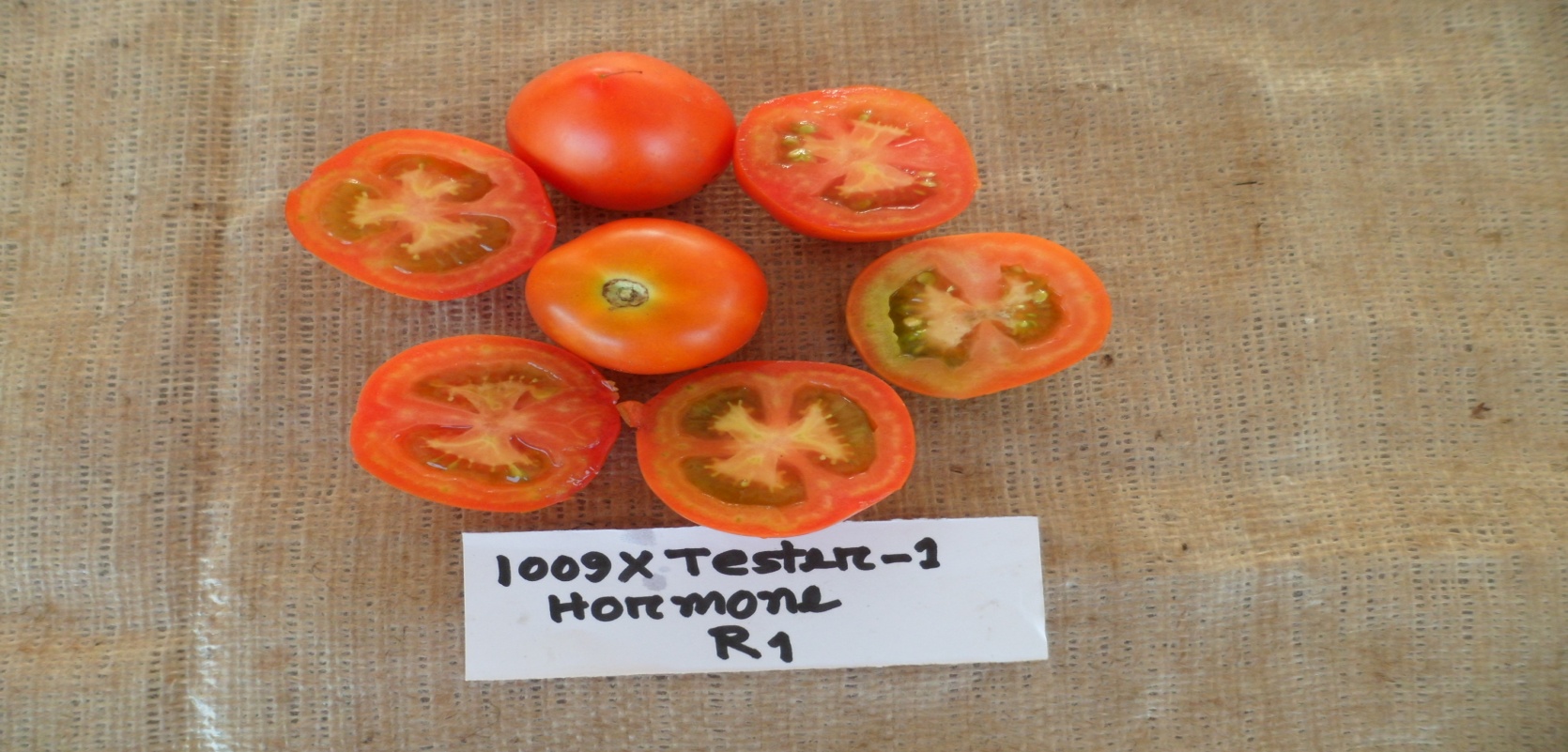 **AVTOV1009 × C41** 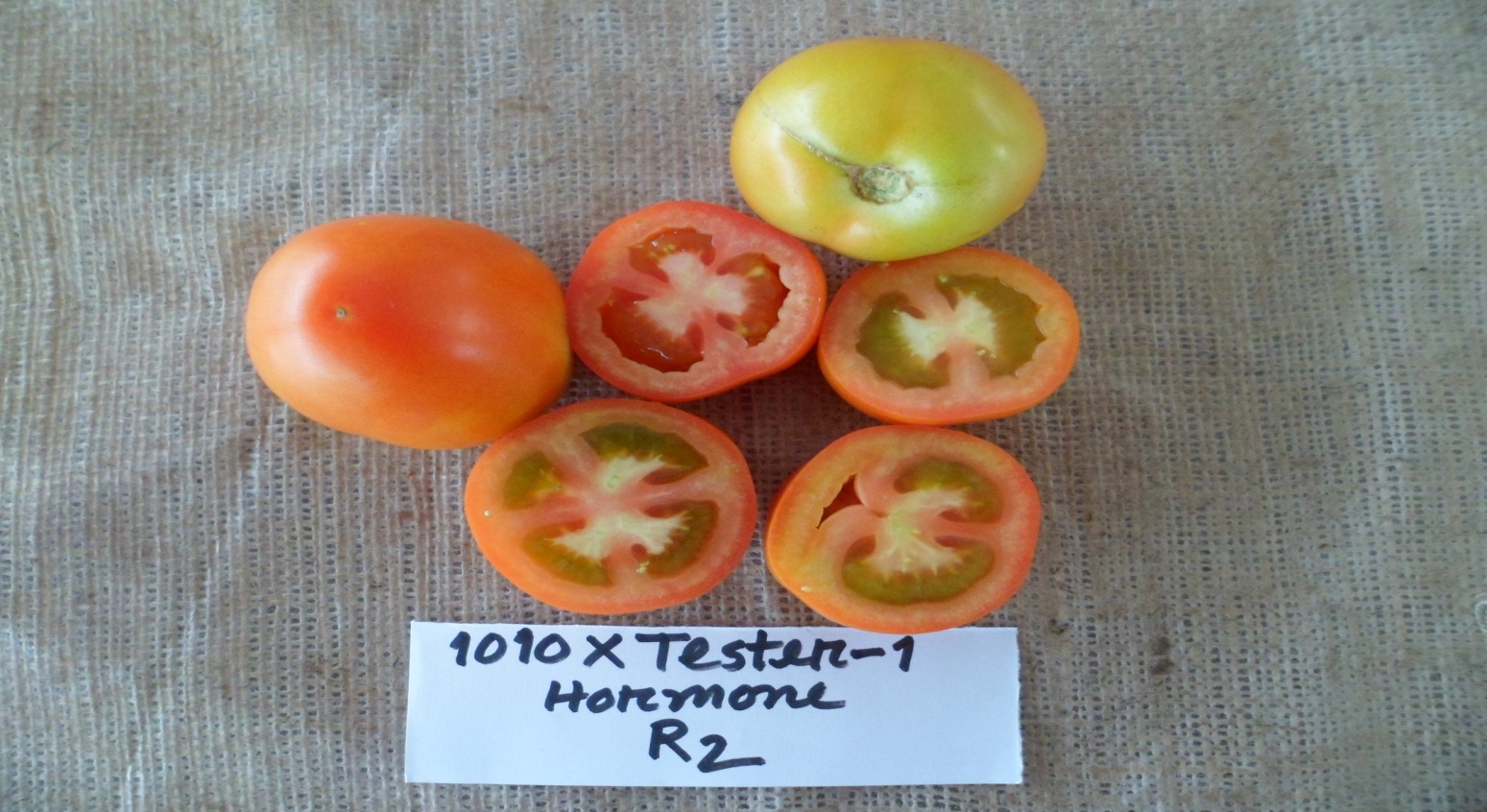 **AVTOV1010 × C41** 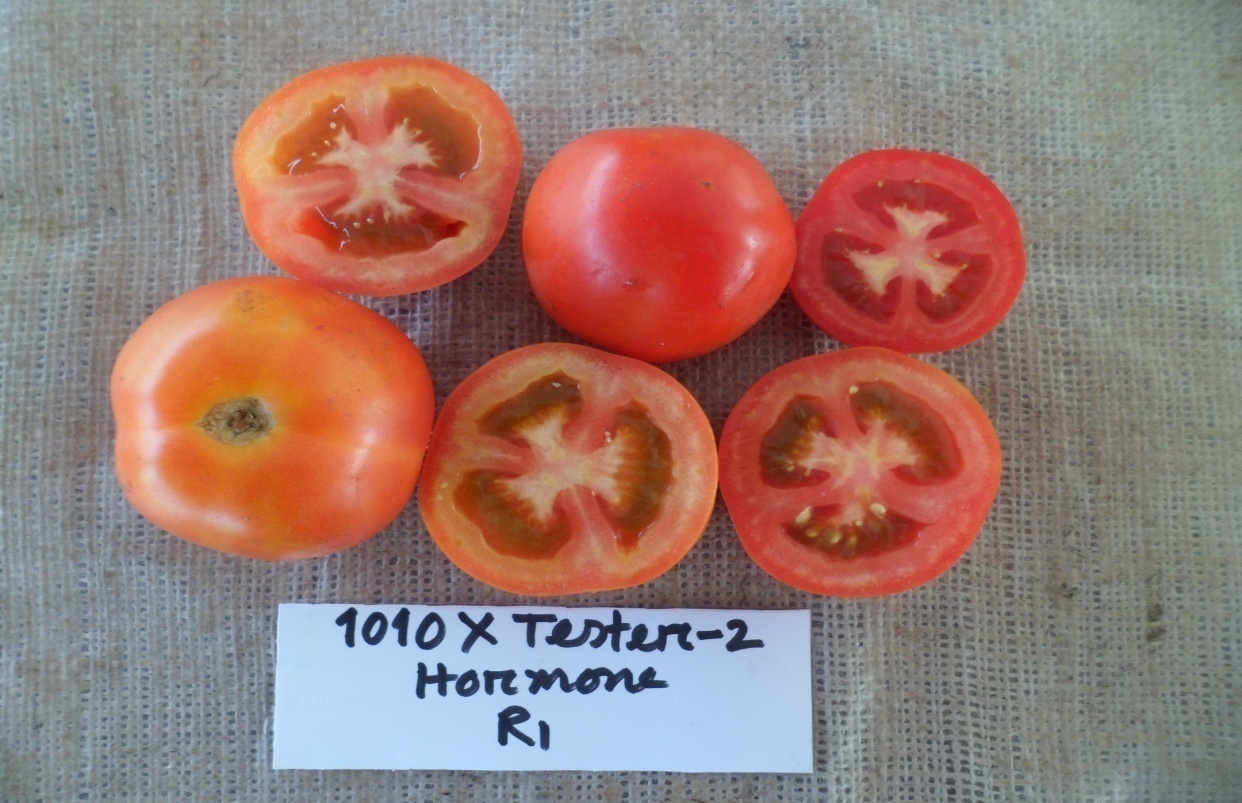 **AVTOV1010 × BARI-4** 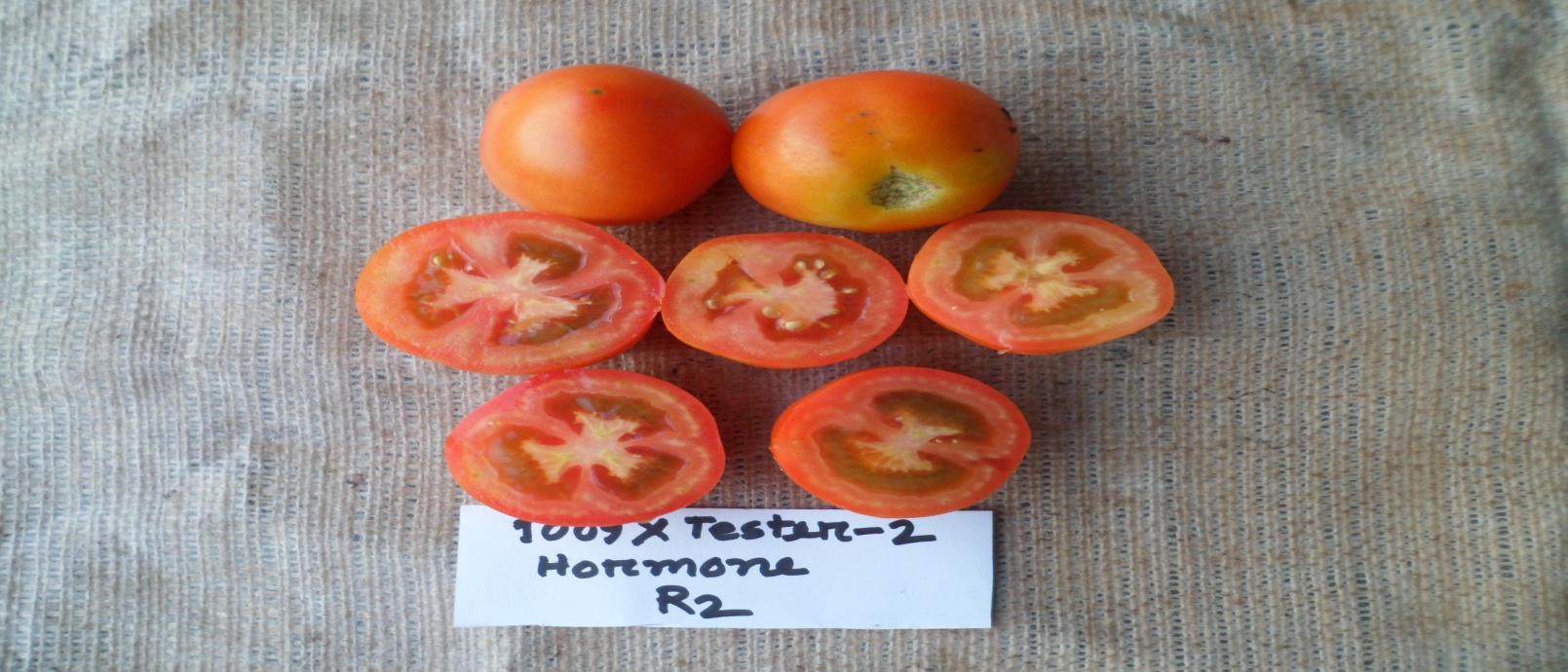 **AVTOV1009 × BARI-4** |  |
| --- | --- |
|  |  |
|  |  |
|  |  |

Fig. 3(b). Fruits of summer tomato hybrids

**Supplementary Table 1. Monthly average Temperature, Relative Humidity and Total rainfall of the experimental site during May to November 2012.**

| **Month** | **Air Temperature (^o^C)** | | **Relative humidity (%)** | | **Total rainfall (mm)** |
| --- | --- | --- | --- | --- | --- |
|  | **Max.** | **Min.** | **Max.** | **Min.** |  |
| **May** | 34.3 | 24.3 | 77.9 | 75.8 | 137 |
| **June** | 33.0 | 26.3 | 78.7 | 73.6 | 185 |
| **July** | 32.3 | 26.4 | 87.7 | 81.3 | 344 |
| **August** | 32.6 | 26.5 | 82.9 | 79.5 | 258 |
| **September** | 32.3 | 26.2 | 92.2 | 82.1 | 180 |
| **October** | 32.5 | 24.2 | 73.8 | 63.1 | 171 |
| **November** | 30.4 | 19.3 | 93.4 | 69.0 | 08 |

Source: Government of the People’s Republic of Bangladesh, Ministry of Defence, Bangladesh Meteorological Department, (Agro-meteorology), Agro-meteorology Pilot Observation Centre, Joydebpur, Gazipur-1701.
